# Advances in Carbon Dot-Based Ratiometric Fluorescent Probes for Environmental Contaminant Detection: A Review

**DOI:** 10.3390/mi15030331

**Published:** 2024-02-28

**Authors:** Xinxin Xing, Zhezhe Wang, Yude Wang

**Affiliations:** 1 School of Materials and Energy, Yunnan University, Kunming 650504, China; zzwang@mail.ynu.edu.cn; 2 Yunnan Key Laboratory of Carbon Neutrality and Green Low-Carbon Technologies, Yunnan University, Kunming 650504, China

**Keywords:** carbon dots, ratiometric fluorescence, environmental monitoring, contaminant detection

## Abstract

Detecting environmental contaminants is crucial for protecting ecosystems and human health. While traditional carbon dot (CD) fluorescent probes are versatile, they may suffer from limitations like fluctuations in signal intensity, leading to detection inaccuracies. In contrast, ratiometric fluorescent probes, designed with internal self-calibration mechanisms, offer enhanced sensitivity and reliability. This review focuses on the design and applications of ratiometric fluorescent probes based on CDs for environmental monitoring. Our discussion covers construction strategies, ratiometric fluorescence principles, and applications in detecting various environmental contaminants, including organic pollutants, heavy metal ions, and other substances. We also explore associated advantages and challenges and provide insights into potential solutions and future research directions.

## 1. Introduction

Environmental contamination, arising from a multitude of sources such as industrial processes, urbanization, and agricultural activities, poses a significant concern for the planet’s well-being [1]. This contamination includes a broad range of hazardous substances, such as organic pollutants, heavy metal ions, and biological species, each presenting unique challenges and threats to ecosystems and public health. Efforts to address environmental contamination hinge on the crucial necessity for precise and efficient detection and monitoring methods. Existing approaches, such as inductively coupled plasma mass spectrometry (ICP-MS) [2,3,4], atomic absorption spectrometry (AAS) [5,6,7,8], high-performance liquid chromatography (HPLC) [9,10,11], and surface-enhanced Raman spectroscopy (SERS) [12,13], have limitations including variable selectivity, sensitivity, and, in some cases, cumbersome analytical procedures. These conventional techniques often require complex sample preparation, time-consuming procedures, and expensive instrumentation, making them less suitable for real-time monitoring and in situ applications. The demand for more advanced and reliable techniques has never been more urgent, especially given the growing global environmental challenges. This urgency necessitates innovative solutions that can address the limitations of existing methods and provide accurate, sensitive, and practical means of environmental monitoring.

Ratiometric fluorescent probes play a crucial role in environmental monitoring, offering precise and reliable detection of various contaminants. Several ratiometric technologies are available, including Fluorescence Resonance Energy Transfer (FRET) [14], Chemiluminescence (CL) [15], Photoacoustic (PA) [16], Bioluminescence (BL) [17], and Afterglow [18], each with its own set of advantages and limitations. For example, FRET provides high sensitivity and resolution but requires matching fluorescent moieties and operates within limited distance ranges. CL, on the other hand, offers high sensitivity and rapid detection but may suffer from background signals and limited specificity. PA imaging offers deep tissue penetration and high resolution but may be hindered by slow imaging speeds and signal attenuation. BL is advantageous for its label-free detection and suitability for in vivo imaging but faces challenges with background noise and tissue penetration depth. Afterglow technology provides long-lasting signals for prolonged tracking and in vivo imaging but may lack sensitivity and resolution.

In contrast, carbon dot (CD)-based ratiometric fluorescent probes offer a promising alternative. Their dual emission peaks enable inherent self-calibration, ensuring precise and selective detection of environmental contaminants [19,20,21,22,23,24]. This property stems from the unique structural and surface characteristics of carbon dots, which facilitate efficient photon emission through mechanisms like quantum confinement and surface passivation [25]. The broad emission spectrum of CDs allows for fluorescence emission across various wavelengths, enhancing their versatility in detecting different analytes with diverse fluorescence properties [26,27]. Furthermore, CDs demonstrate remarkable fluorescence stability under diverse environmental conditions, such as exposure to light, temperature changes, and chemical environments. This stability ensures reliable and consistent fluorescence signals during environmental monitoring applications. The multiple emissive centers on the surface of CDs contribute to their unique emission peaks, providing a foundation for precise and selective detection of various contaminants [28,29]. Additionally, factors like pH [30], solvent polarity [31], and surface functionalization [32] can modulate the fluorescence intensity of CDs, further expanding their applicability in environmental sensing. CDs’ dual emission peaks serve as an intrinsic self-calibration mechanism, offering precise and selective detection of various contaminants [33,34,35,36,37,38,39,40,41,42,43]. This unique feature positions CDs as promising candidates for advanced environmental contaminant detection, offering a solution to the limitations of traditional fluorescent probes. However, CD-based ratiometric fluorescent probes, while promising, also have limitations. These may include issues related to photobleaching, signal stability, and potential interference from background fluorescence, necessitating further investigation and optimization.

Despite numerous studies [7,44,45,46,47,48,49,50,51,52,53,54,55,56] and reviews [57,58,59,60,61,62,63] on CD-based ratiometric fluorescent probes, a comprehensive review dedicated to their role in environmental contaminant detection is notably absent. In this review, we highlight the unique potential of ratiometric fluorescent probes based on CDs for environmental monitoring. We first explore their construction strategies and the underlying principles of ratiometric fluorescence. Our discussion encompasses their diverse applications in detecting various environmental contaminants, including organic pollutants, heavy metal ions, and other contaminants. We also delve into the associated benefits and challenges, emphasizing the need for improved selectivity, sensitivity, and real-time monitoring. Additionally, we offer insights into potential solutions and future research directions, considering aspects such as probe stability and expanding analyte detection capabilities. Through this comprehensive review, we aim to contribute to the promotion of environmental safety and the advancement of sensing technology.

## 2. Construction Strategies of CDs in Detecting Environmental Contaminants

The construction of ratiometric fluorescent probes based on CDs can be categorized into various strategies, including surface modification, integration of composite materials, simple mixing strategy, and dual emission techniques. This section provides a comprehensive overview of how each strategy contributes to enhancing the performance of CDs in detecting environmental contaminants.

### 2.1. Surface Modification Strategy

Surface modification is a fundamental approach to enhance the properties of CDs to meet the specific requirements of environmental contaminant detection. By strategically altering CD surfaces, such as optimizing surface charge to enhance aqueous dispersibility or attaching specific ligands to target particular contaminants, surface modification provides a versatile means to fine-tune CDs for optimal performance. This adaptability makes surface modification a cornerstone strategy, enabling researchers to effectively address solubility, selectivity, and stability issues.

**Figure 1:**
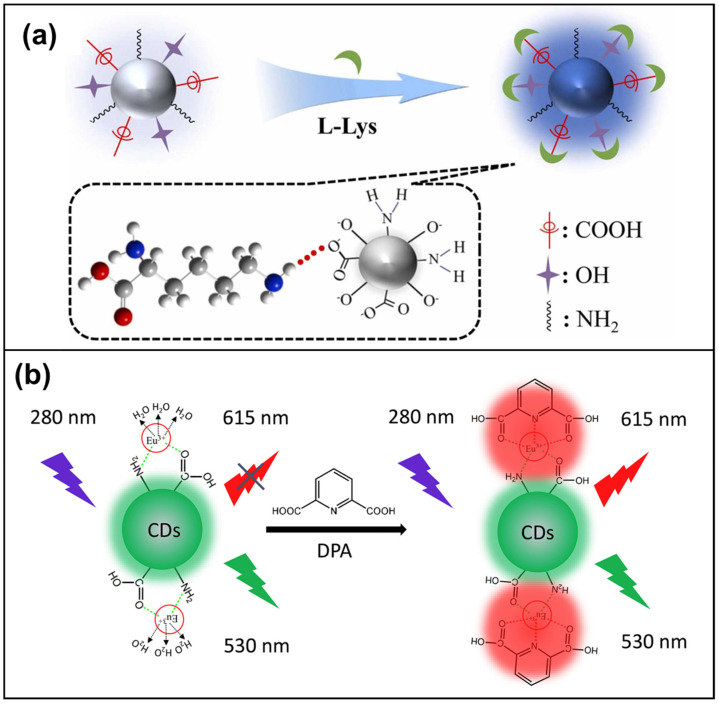
(**a**) Schematic of proposed route for carboxyl-, hydroxyl-, and amino groups-functionalized CDs for the detection of L-Lysine [64]; (**b**) schematic illustration of the carboxyl- and amino groups-functionalized CD probe for the detection of DPA [75].

Chemical surface modification introduces various functional groups onto CD surfaces to enhance solubility, reactivity, and specific ligand conjugation [64,65,66,67,68,69,70,71,72,73] (Figure 1a). For instance, Xu et al. [74] reported a ratiometric CD pH sensor enriched with amino groups, enabling them to specifically target lysosomes within living cells. The CDs were functionalized with abundant amino groups during their synthesis, exhibiting dual emission bands at 439 and 550 nm under single-wavelength excitation without the need for additional labeling. This sensor demonstrates robust lysosomal targeting, as evidenced by high Pearson’s colocalization coefficients (0.935 and 0.924), which indicate the degree of spatial overlap between the fluorescent signals emitted by the CDs and the lysosomal markers. Wang et al. [75] developed a novel ratiometric fluorescent probe for dipicolinic acid (DPA) point-of-care testing (POCT) by functionalizing CDs containing carboxyl and amino groups with Eu(III) ions (CDs-Eu) (Figure 1b). This approach features an exceptional detection limit of 0.8 nM.

Biological surface modification utilizes biomolecules like proteins, DNA, and enzymes to modify CDs, enabling the creation of highly selective probes for specific environmental pollutants or biological markers. Bu et al. [76] combined red-emitting DNA-templated copper nanoclusters (CuNCs) with blue-emitting CDs, forming a self-assembled complex known as DNA-CuNC/CDs through electrostatic forces. This complex serves as a dually emitting ratiometric probe for simultaneously detecting arginine and acetaminophen, with detection limits of 0.35 μM and 0.26 μM, respectively. Hu et al. [77] introduced a straightforward technique for fabricating vesicle-like CDs (VCDs) by dry heating surfactant solutions. Similar to many previously reported CDs, these VCDs display intriguing fluorescence properties. The incorporation of enzymes and gold nanoclusters (AuNCs) within the VCDs enables the development of fluorescent probes for quantifying diverse substrates, offering significant advantages in sensitivity and selectivity.

### 2.2. Composite Strategy

The composite strategy involves integrating CDs with other nanomaterials like metal nanoparticles, quantum dots, or other nanomaterials, to enhance their performance as fluorescent probes. This integration occurs through chemical bonding or interactions, resulting in a new composite material. Consequently, these composite probes display improved fluorescence characteristics, greater stability, and heightened sensitivity, making them valuable tools for environmental contaminant detection and other analytical applications.

**Figure 2:**
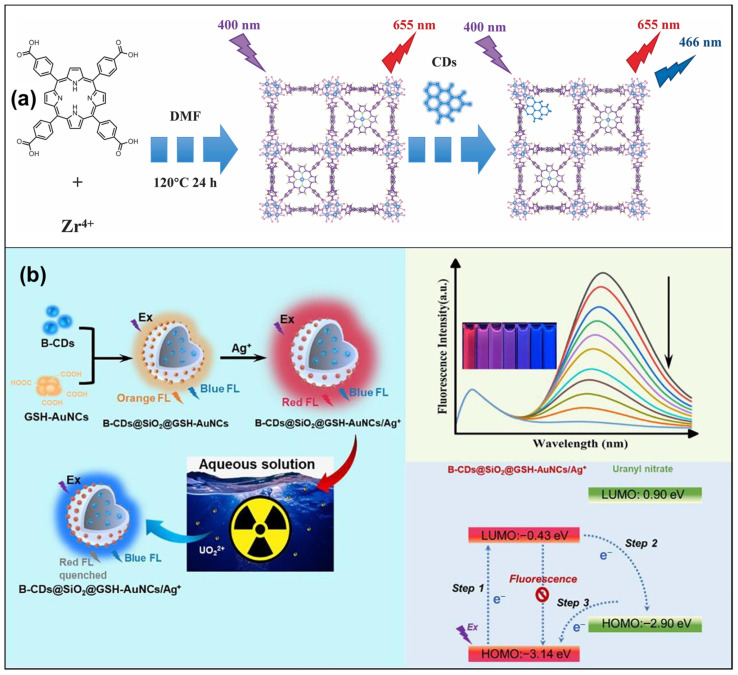
(**a**) Process for the solvothermal synthesis of PCN-224 and the PSM of PCN-224 with CDs [79]; (**b**) the preparation process of B-CDs@SiO_2_@GSH-AuNCs/Ag^+^ and the schematic diagram of the detection of UO_2_^2+^ [80].

A commonly employed strategy involves amalgamating CDs with other optical materials featuring unsaturated sites [78,79]. For instance, Zhou et al. [79] synthesized the CDs-PCN-224 (porphyrin-based metal-organic framework nanoparticles) fluorescent probe by post-synthetically modifying luminescent metal-organic frameworks and CDs. They combined the abundant groups on the surface of CDs with the unsaturated sites provided by the activated PCN-224 (Figure 2a). In this probe, the fluorescence of CDs served as a reference signal, while the fluorescence of PCN-224 acted as a highly sensitive response signal with active sites designed for detecting copper ions. The CDs-PCN probe exhibited rapid, sensitive, and selective response to copper ions, boasting an impressively low detection limit of 44 nM.

A core-shell structure is also a frequently adopted approach to protect the sensing capabilities of CDs fluorescent probes [80,81,82,83,84,85]. For example, Gong et al. [80] reported a novel and stable ratiometric fluorescent probe (B-CDs@SiO_2_@GSH-AuNCs/Ag^+^) designed for the sensitive detection of uranyl in water (Figure 2b). This probe exhibited exceptional stability, with the *F*/*F*_440_ ratio decreasing by merely 4.73% even after three months in a chloroacetic acid-sodium acetate buffer solution (pH 3.0).

### 2.3. Simple Mixing Strategy

**Figure 3:**
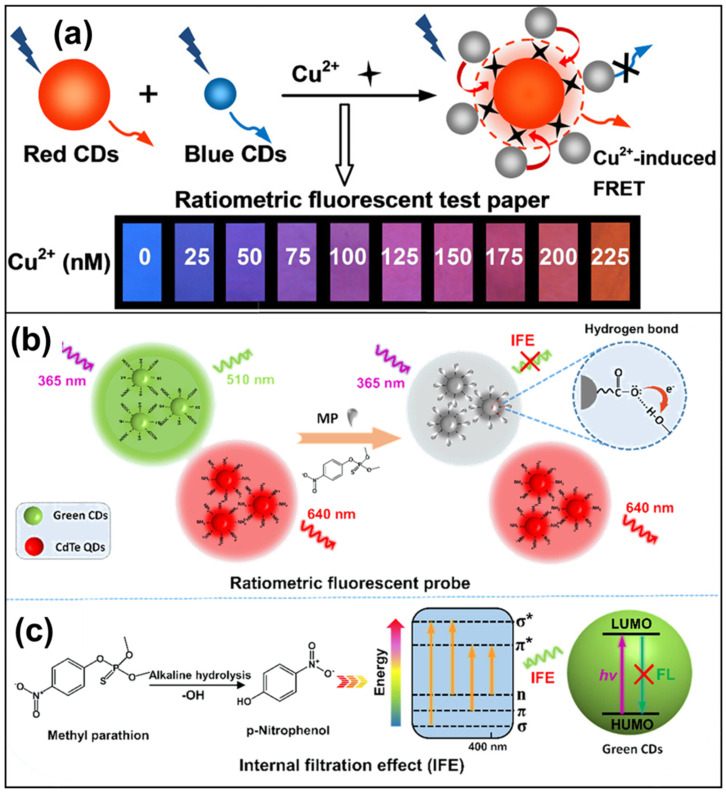
(**a**) Dual-colored CD ratiometric fluorescent test paper for the semiquantitative assay of Cu^2+^ [92]; (**b**) integration of green CDs and CdTe quantum dots for highly selective quantitative detection of MP [88]; (**c**) sensing mechanism of internal filtration effect (IFE) of p-NP to green CDs (* represents the excited state) [88].

The simple mixing strategy involves the direct physical combination of CDs with other components without covalent bonding or strong interactions between them. This method offers a rapid and convenient approach to creating ratiometric fluorescent probes for environmental contaminant detection. CDs are physically mixed with other reference luminescent materials, such as polymers, nanoparticles, or other fluorophores, to establish a dual-emission system [67,86,87,88,89,90,91,92,93,94,95,96,97] (Figure 3a).

The adaptability of the simple mixing strategy facilitates the creation of ratiometric fluorescent probes tailored for various environmental contaminant detection scenarios. Through the selection of suitable companion materials and precise adjustment of mixing ratios, this method can be personalized to target specific analytes or adapt to a broad spectrum of environmental conditions. Additionally, the resulting probes can often be produced rapidly, making them attractive for applications requiring rapid response times, such as on-site monitoring and point-of-care testing. Ghasemi et al. [86] devised a ratiometric fluorescent probe composed of blue-emissive CDs (BCDs) in combination with thioglycolic acid (TGA)-capped yellow-emissive cadmium telluride (CdTe) quantum dots (YQDs). This probe demonstrates dual emissions, peaking at 443 and 560 nm, with a single excitation wavelength of 360 nm. Upon exposure to Hg(II) ions, as a representative example, the fluorescence of the YQDs undergoes selective quenching and redshift, leading to a continuous change in the probe’s emission color. This transition ranges from vibrant green to lighter shades of green, yellow-green, and yellow, eventually shifting towards warmer tones such as orange, pink, purple, weak blue, and even dark blue. The fluorescent ratiometric probe exhibits an impressively low detection limit of 4.6 nM. Qin et al. [88] developed a ratiometric fluorescence probe that combines green CDs with CdTe QDs, enabling highly selective and quantitative detection of methyl parathion (MP) (Figure 3b,c). Under alkaline conditions, MP undergoes rapid hydrolysis, yielding p-nitrophenol (p-NP). This immediate chemical transformation triggers the reinforcement of hydrogen bonds, creating an internal filter effect between the CDs and p-NP. Consequently, this interaction quenches the green fluorescence, resulting in a vivid and instant transition from green to red emission. The probe demonstrates an impressive level of sensitivity, with a detection limit as low as 8.9 nM.

### 2.4. Dual Emission Strategy

**Figure 4:**
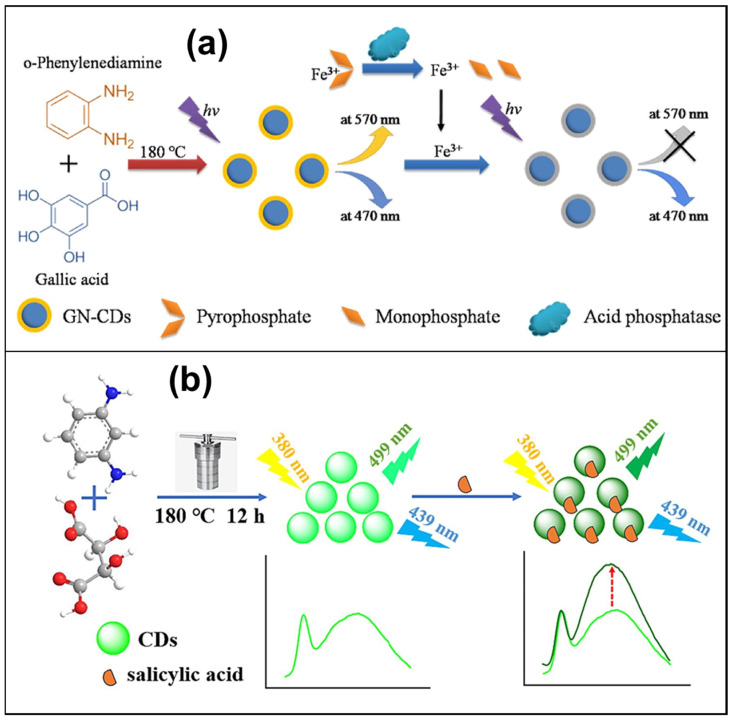
(**a**) Gallic acid and o-phenylenediamine were used as raw materials to prepare dual-emission CDs exhibiting two fluorescent emission peaks at 470 and 570 nm, respectively [104]; (**b**) the CDs, synthesized using tartaric acid (TA) and m-phenylenediamine (mPD) through a straightforward one-step hydrothermal method, display two fluorescence emission peaks at 499 nm and 439 nm when excited at 380 nm [105].

The dual emission strategy is a versatile and effective approach that capitalizes on the intrinsic fluorescence properties of CDs. In this method, CDs are engineered or modified to emit light at two distinct wavelengths under the excitation of a single wavelength [64,66,67,74,98,99,100,101,102,103,104,105,106,107] (Figure 4). These dual-emitting CDs offer a built-in reference signal, enhancing the accuracy and reliability of ratiometric measurements. The concept behind dual-emitting CDs involves generating two distinct fluorescence signals within a single nanomaterial. One of these emissions typically serves as the analyte-specific response signal, while the other serves as a constant reference signal. The reference signal remains unaffected by environmental changes, making it an ideal internal standard for calibration and quality control [64,67,103,104,105,106,107].

This built-in reference signal significantly enhances the accuracy and reliability of ratiometric measurements in detecting environmental contaminants. It helps alleviate potential sources of error, such as fluctuations in excitation intensity or variations in sample matrix composition. Furthermore, the dual emission strategy allows for real-time monitoring of changes in analyte concentration or environmental conditions, making it particularly advantageous for on-site and in situ applications. Paydar et al. [98] modified CDs through a simple chemical surface modification using glutathione. The modified CDs displayed a unique spectral profile characterized by two distinctive emission peaks, separated by 170 nm, ranging from 522 to 692 nm. This probe not only enabled precise quantification of Pb^2+^ but also exhibited remarkable sensitivity, with a detection limit of Pb^2+^ as low as 2.7 nM. Chen et al. [99] introduced a near-infrared ratiometric fluorescent probe based on nitrogen and sulfur co-doped CDs (N, S-CDs). This probe was synthesized using a hydrothermal approach, employing glutathione and formamide as precursors. The N, S-CDs, due to their nitrogen and sulfur atom doping, readily form complexes with Zn^2+^. Under excitation at 415 nm, the ratio (I_650_/I_680_) of fluorescence intensity at 650 nm to 680 nm exhibited a direct correlation with the concentrations of Zn^2+^. The probe demonstrated a remarkable detection limit of 5.0 nM for Zn^2+^.

## 3. Application in Environmental Monitoring

Detecting environmental contaminants presents unique challenges due to the diverse nature of pollutants and their potential harm to ecosystems and human health. Traditional analytical methods often lack the sensitivity, selectivity, and real-time monitoring capabilities needed for effective environmental assessment. The application of ratiometric fluorescence principles in contaminant detection using CDs has emerged as a powerful analytical approach. CDs’ ratiometric fluorescence offers a compelling solution by leveraging the inherent optical properties of CDs to address these challenges. In this approach, CDs are strategically modified or functionalized to interact specifically with target pollutants. The presence of environmental contaminants triggers changes in the CD emission properties, leading to alterations in dual-emission characteristics. These changes form the basis for ratiometric measurements, enabling the quantification of contaminant levels and differentiation of target pollutants from potential interferents. This method has practical applications in various environmental monitoring scenarios, including the detection of organic pollutants, heavy metals, and other harmful substances.

### 3.1. Heavy Metal Ion Sensing

The detection and quantification of heavy metal ions in the environment are crucial due to their high toxicity and potential adverse effects on ecosystems and human health. Heavy metal ions, such as mercury (Hg^2+^) [86,101,108,109,110,111,112,113,114,115], lead (Pb^2+^) [67,98,106,116,117,118,119], chromium (Cr^6+^) [120,121,122,123], copper (Cu^2+^) [67,79,92,109,119,124,125,126,127,128,129,130], and silver (Ag^+^) [78,117,131,132,133], can contaminate water, soil, and air through various industrial processes and human activities. Table 1 summarizes sensing of some heavy metal ions using CD-based ratiometric fluorescence probes. CDs have proven to be versatile tools for the sensitive and selective detection of heavy metal ions. The application of CD-based ratiometric fluorescence probes in heavy metal ion sensing relies on specific interactions between CDs and metal ions, resulting in changes in the CD emission properties.

The mechanisms underlying the detection of heavy metal ions using CD-based ratiometric fluorescence probes vary depending on the specific interactions between CDs and the metal ions. These interactions can be broadly categorized into four mechanisms: chelation, surface modification, quenching, and aggregation-induced changes.

**Figure 5:**
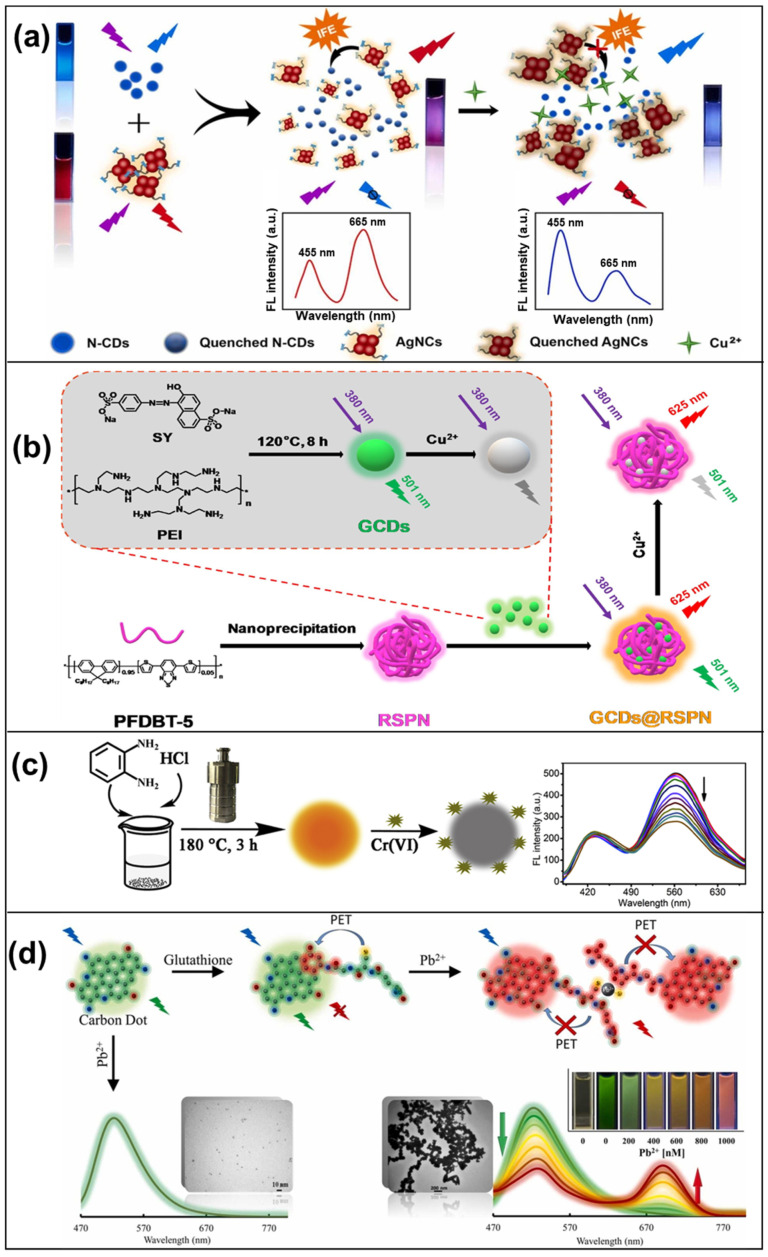
(**a**) Upon the introduction of Cu^2+^, the red fluorescence of AgNCs diminishes due to the robust chelating interaction between L-glutathione and Cu^2+^, while the blue fluorescence undergoes a resurgence, leading to a notable shift in fluorescence color from pink to blue under UV light [124]; (**b**) a dual-emissive fluorescent ratiometric probe for Cu^2+^ was developed by loading amine-coated CDs onto red emission semiconducting polymer nanoparticles (RSPN) through electrostatic adsorption [127]; (**c**) schematic illustration of Y-CDs preparation and the quenching process upon the addition of Cr(VI) to the system [121]; (**d**) the average dynamic size of m-AP-GSH CDs transformed from 1.67 nm without Pb^2+^ to 289.3 nm after Pb^2+^ addition, indicating significant aggregation of the CDs with Pb^2+^ [98].

Functionalized CDs with specific ligands or receptors have the unique ability to form stable complexes with heavy metal ions through chelation. This involves binding metal ions to functional groups on the CD surface, creating enduring chemical bonds (Figure 5a). As a result, this chelation process induces significant alterations in the CDs’ emission properties, allowing for precise detection of metal ions such as Hg^2+^ [112], Pb^2+^ [106], and Cu^2+^ [124]. This mechanism is widely used in environmental monitoring, especially in assessing water and soil quality due to its high sensitivity and selectivity.

Surface modification techniques serve as a versatile approach to engineer CDs for the selective adsorption of heavy metal ions. By leveraging various methods, including electrostatic interactions, ion exchange, or chemical binding, CDs can be tailored to adsorb specific metal ions (Figure 5b). This surface modification technique results in the absorption of metal ions onto the CD surface, thereby altering the CD’s photophysical properties. These changes enable ratiometric measurements for the detection of metal ions such as Pb^2+^ [98] and Cu^2+^ [109,127], which are frequently detected using this mechanism.

In certain situations, heavy metal ions directly quench the fluorescence of CDs. This quenching phenomenon arises from the binding of metal ions to CDs, causing a non-radiative energy transfer that reduces the fluorescence intensity of the CDs (Figure 5c). As a result, this quenching mechanism is employed for the detection of a wide range of metal ions, including Hg^2+^ [86,101,108,109,112], Ag^+^ [131,132], Cr^6+^ [120,121,122], and Cu^2+^ [67,92,126]. Its applicability extends to various fields, including clinical diagnostics and environmental monitoring, due to its simplicity and ability to yield real-time results.

The presence of heavy metal ions can induce the aggregation of CDs through interactions with the metal ions, a phenomenon often described as aggregation-induced changes. This process leads to noticeable alterations in the photophysical properties of CDs (Figure 5d). Specifically, it results in changes in the dual-emission characteristics that are used for ratiometric measurements. As such, this mechanism has found significant application in the detection of metal ions such as Cu^2+^ [67,109,124,129] and Pb^2+^ [67,98,116]. Its unique sensitivity to changes in the aggregation state of CDs has made it a valuable tool in the study of complex environmental samples and biological systems.

### 3.2. Detection of Organic Pollutants

Detecting organic pollutants accurately and precisely is crucial for environmental monitoring because these contaminants consist of various compounds, each with distinct chemical properties and potential environmental and health impacts. Organic pollutants encompass polycyclic aromatic hydrocarbons (PAHs), pesticides, herbicides, pharmaceutical residues, industrial chemicals, and other recalcitrant organic compounds. Assessing environmental quality and ensuring public safety heavily relies on the detection and quantification of these substances.

**Figure 6:**
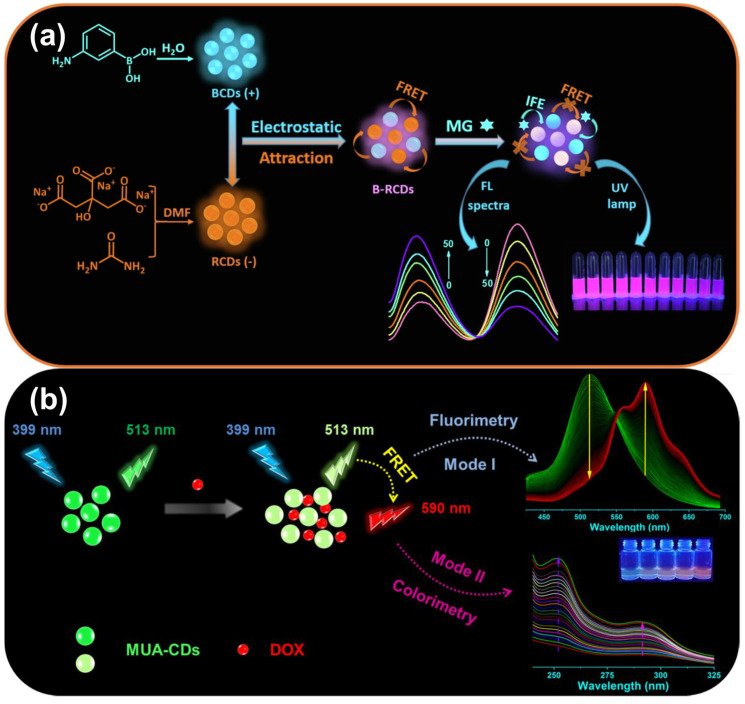
(**a**) Schematic diagram of construction method for blue/red-emission CDs probe and ratiometric response to malachite green [135]. (**b**) Illustration of 11-mercaptoundecanoic acid-functionalized CDs as a ratiometric fluorescence probe for selective doxorubicin (DOX) detection. Mode I displays the fluorescence spectra of mercaptoundecanoic-CDs (MUA-CDs) (0.2 mg/mL) under excitation at 399 nm, responding to varying DOX concentrations (0.25–97.07 μM). Mode II exhibits the UV-vis absorption spectra of MUA-CDs under different DOX concentrations (0–63.20 μM) [148].

Pesticides and herbicides pose significant challenges in environmental monitoring, and CDs offer a promising solution for their detection and sensing [134,135,136] (Figure 6a). Shokri et al. [134] developed a novel method to detect triticonazole using a dual-emission ratiometric fluorescence sensor. This sensor involved encapsulating boron-doped CDs (B-CDs) with blue fluorescence and phosphorus-doped green-emitting CDs (P-CDs) into a zeolitic imidazolate framework-8 (ZIF-8). The B-CDs/P-CDs@ZIF-8 composite displayed two distinct emission peaks at 440 nm and 510 nm when excited at a single wavelength of 385 nm, corresponding to B-CDs and P-CDs, respectively. In the presence of triticonazole, the fluorescence intensity of B-CDs decreased significantly, while that of P-CDs remained constant. As the concentration of triticonazole increased, the color of the ratiometric probe shifted gradually from blue to green. Under optimized conditions, the B-CDs/P-CDs@ZIF-8 probe exhibited a low detection limit of 4.0 nM for triticonazole.

Pharmaceutical residues can disrupt ecosystems, contribute to antibiotic resistance, and lead to various health issues, including allergies and poisoning. Monitoring and controlling pharmaceutical residues are crucial to mitigate these risks [65,69,81,88,105,137,138,139,140,141,142,143,144,145,146,147,148,149,150] (Figure 6b). One fundamental mechanism in detecting pharmaceutical residues is related to electron transfer processes. Adsorption of pharmaceutical residues onto the surfaces of CDs can trigger electron transfer processes, resulting in alterations in the CDs’ emission properties. Jalili et al. [141] introduced a rapid-response ratiometric probe for the sensitive visual detection of the banned veterinary antibiotic chloramphenicol (CLP), which is still illicitly used in animal husbandry. They utilized two kinds of CDs, one with yellow emission (Y/CDs, 560 nm) as the target-sensitive component and the other with blue emission (B/CDs, 440 nm) as reference dyes to create the ratiometric fluorescence probe (mMIP@YBCDs). In the presence of CLP, interactions between amino groups (-NH_2_) in the APTES molecule, located within the binding sites of mMIP@YBCDs, and functional groups in CLP, such as the carbonyl and nitro groups, lead to the formation of a Meisenheimer complex through hydrogen bonding. This interaction results in electron transfer between Y/CDs and CLP, significantly inhibiting radiative recombination of the electron–hole pair. Consequently, the majority of excited electrons return to the ground state via nonradiative decay instead of radiative decay, causing a reduction in fluorescence intensity.

### 3.3. Other Contaminants

In addition to organic pollutants and heavy metal ions, CD-based ratiometric fluorescence probes have been utilized in detecting various other environmental contaminants. These contaminants consist of a diverse range of substances, each presenting unique challenges for detection and quantification. The versatility and adaptability of CD-based probes make them well-suited for addressing these challenges.

Anions: CD-based probes have been employed for detecting various anions, including phosphate (Pi) [87,103,151], nitrite (NO_2_^−^) [68,152], hypochlorite (ClO^−^) [153,154], bisulfite [155], and sulfide (S^2−^) [156] in water sources, facilitating the assessment of water quality.

**Figure 7:**
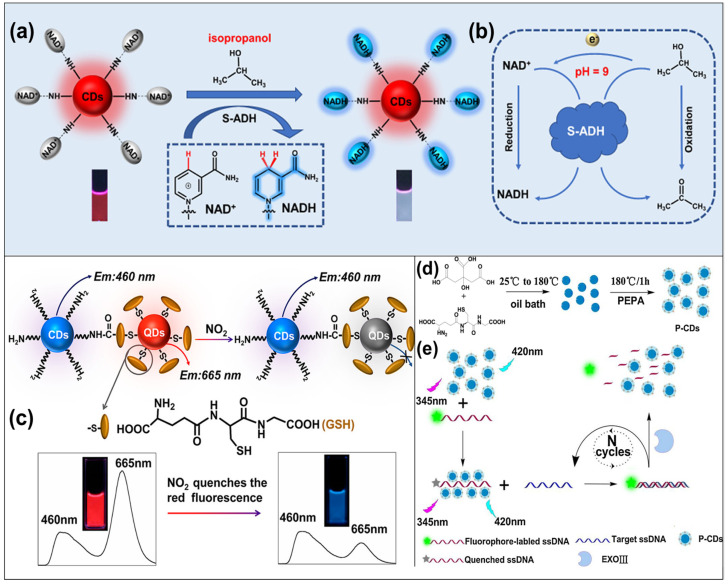
(**a**) Schematic diagram of the ratiometric fluorescence sensing system for isopropanol detection and (**b**) reaction mechanism of the probe sensing part nicotinamide adenine dinucleotide (NAD^+^) for isopropanol [161]; (**c**) schematic illustration of blue-emission CDs and red-emission CdTe QDs hybrid probe structure and the visual detection principle for NO_2_ [162]; (**d**) synthesis of positive-charged CDs (P-CDs) in a two-step procedure; (**e**) the quenching of fluorophore-labelled ssDNA by P-CDs, while retaining the stable fluorescence intensity of P-CDs, enabling a ratiometric analytical method for gardnerella vaginalis DNA with the target sequence circulating under the assistance of exonuclease III (Exo III) [168].

Gases and Vapors: CD-based sensors have been developed for detecting gases such as oxygen (O_2_) [157], hydrogen sulfide (H_2_S) [66,158], volatile organic compounds (VOCs) [122,159,160,161] (Figure 7a,b), and nitrogen dioxide (NO_2_) [162] (Figure 7c) in liquid or air quality monitoring scenarios.

Biological and Biomolecular Targets: CD-based ratiometric fluorescence probes have been adapted for detecting specific biological and biomolecular targets, such as bacteria [82,163,164], biomarkers [75,165], cholesterol [77], amino acids [64], guanine [166,167], DNA [168] (Figure 7d,e), and proteins [1,91,107,169,170]. These probes find applications in genomics, molecular diagnostics, and bioanalytical assays.

## 4. Conclusions and Outlook

Detecting environmental contaminants is crucial for safeguarding ecosystems and public health. While traditional CD fluorescent probes have shown versatility in environmental monitoring, they are not without limitations, particularly in terms of the accuracy and reliability of detection results due to fluctuations in absolute signal intensity. Ratiometric fluorescent probes, engineered with internal self-calibration mechanisms, offer significant advantages, including enhanced sensitivity and reliability. In this review, we have explored the design and applications of ratiometric fluorescent probes based on CDs for environmental monitoring. Our discussion has covered construction strategies, ratiometric fluorescence principles, and applications in detecting various environmental contaminants, including organic pollutants, heavy metal ions, and other environmental threats. Additionally, we have outlined future directions as follows:

- Sustainable synthesis of biocompatible CDs for eco-friendly sensing: Most CDs are made using non-renewable materials and energy-intensive methods, which harm the environment. Additionally, biocompatible CDs are a new area with limited practical uses. Creating biocompatible CDs supports the global shift towards green chemistry and sustainability. These CDs can reduce environmental risks, making sensing applications safer. Their use also cuts down on hazardous waste and promotes renewable resources.
- Multiplexed sensing for comprehensive environmental analysis: Currently, many CD-based ratiometric probes are designed for single analytes, which makes combining multiple sensing capabilities into one probe challenging due to possible cross-reactivity. Since the environment usually has a mix of contaminants, creating multiplexed ratiometric probes is crucial. These probes can detect various contaminants simultaneously, reducing the need for many sensors. This approach provides a more accurate assessment of environmental conditions by handling the complex nature of contaminants.
- Portable field sensors for real-time monitoring: While CD-based ratiometric sensors are mainly used in labs, their usefulness in field applications is limited. Even portable sensors may lack durability and quick detection capabilities. Portable sensors are important for real-time monitoring in tough environmental conditions, enabling rapid responses to emergencies. Field monitoring ensures timely environmental assessments and responses, especially in remote or disaster-prone areas. Developing portable, strong sensors is vital for improving data collection.
- Microscale environmental mapping for detailed insight: Microscale environmental mapping using CD-based ratiometric fluorescence has limitations in spatial precision and integrating diverse sensing technologies. Many environmental issues require a microscale perspective for deep understanding. Integrated spatial data improve decision-making. Enhanced spatial precision offers detailed insight into contamination patterns. Collaboration across disciplines helps interpret data effectively and derive actionable insights.

## Figures and Tables

**Table 1:** A summary of CD-based ratiometric fluorescence probes for the detection of heavy metal ions.

Ratiometric FL Probes	Construction Strategy	Analyte	LOD	Ref.
ZIF-8@g-CNQD/CdTe	Quench of CdTe	Hg^2+^	~46 nM	[111]
Ag/Au@CDs nanohybrids	Ligand effect	Hg^2+^	7 nM	[109]
YQDs + BCDs	Quench of CdTe	Hg^2+^	4.6 nM	[86]
Dual-emissive CDs	Ligand effect	Hg^2+^	0.27 μM	[101]
(NCDs-RhB@COF	Ligand effect	Hg^2+^	15.9 nM	[108]
CDs and CdSe@ZnS QDs	Quench of CdSe@ZnS QDs	Hg^2+^	0.1 μM	[112]
CDs and Si NCs	Quench of Si NCs	Hg^2+^	7.63 nM	[110]
CuNCs-CNQDs	Aggregation of CuNCs	Pb^2+^	0.0031 mg L^−1^	[116]
N-CDs/AuNCs	Aggregation of AuNCs	Pb^2+^	0.5 μM	[67]
GSH-modified CDs	Aggregation of CDs	Pb^2+^	2.7 nM	[98]
Label-free CDs	Ligand effect	Pb^2+^	0.055 μM	[106]
Y-CDs	IFE	Cr^6+^	2.3 nM	[121]
Dual-emissive CDs	IFE	Cr^6+^	0.4 μM	[120]
N-doped Dual-emissive CDs	IFE	Cr^6+^	3.2 µM	[122]
Ag/Au@CDs nanohybrids	Aggregation of CDs	Cu^2+^	5 nM	[109]
N-CDs/AuNCs	Quench of AuNCs	Cu^2+^	0.15 µM	[67]
N-CDs/AgNCs	Aggregation of N-CDs/AgNCs	Cu^2+^	0.13 µM	[124]
r-CDs and b-CDs (1:7)	Quench of b-CDs	Cu^2+^	8.82 nM	[92]
Dual-emissive CQDs	Ligand effect	Cu^2+^	-	[130]
Dual-emissive N-CDs	Ligand effect	Cu^2+^	17.7 nM	[126]
GCDs@RSPN	Quench of GCDs	Cu^2+^	0.58 μM	[127]
Dual-mode SQD–CQD probe	IFE	Cu^2+^	31 nM and 47 nM	[128]
MPA-CdTe and CDs	Quench of MPA-CdTe	Cu^2+^	0.36 nM	[125]
CDs-PCN	Ligand effect	Cu^2+^	44 nM	[79]
NCCOF_TAPT-TT_	Photoinduced electron transfer	Cu^2+^	17.3 nM	[129]
P-CDs/R-CDs	Quench of P-CDs	Ag^+^	32 nM	[132]
NALC-CdTe QDs and N,Si-CQDs	Quench of NALC-CdTe QDs	Ag^+^	1.7 nM	[131]
CSs-AuNCs	Increased FL of AuNCs	Ag^+^	1.6 nM	[78]

## Data Availability

No applicable.
